# The Relationship Between Childhood Emotional Abuse and Processing of Emotional Facial Expressions in Healthy Young Men: Event-Related Potential and Behavioral Evidence

**DOI:** 10.3389/fpsyg.2021.686529

**Published:** 2021-09-09

**Authors:** Yutong Liu, Huini Peng, Jianhui Wu, Hongxia Duan

**Affiliations:** ^1^Center for Brain Disorder and Cognitive Science, Shenzhen University, Shenzhen, China; ^2^Donders Institute for Brain, Cognition, and Behavior, Radboud University Medical Center, Nijmegen, Netherlands

**Keywords:** childhood emotional abuse, emotion, facial expressions, ERP, P200

## Abstract

**Background:** Individuals exposed to childhood maltreatment present with a deficiency in emotional processing in later life. Most studies have focused mainly on childhood physical or sexual abuse; however, childhood emotional abuse, a core issue underlying different forms of childhood maltreatment, has received relatively little attention. The current study explored whether childhood emotional abuse is related to the impaired processing of emotional facial expressions in healthy young men.

**Methods:** The emotional facial processing was investigated in a classical gender discrimination task while the event-related potentials (ERPs) data were collected. Childhood emotional abuse was assessed by a Childhood Trauma Questionnaire (CTQ) among 60 healthy young men. The relationship between the score of emotional abuse and the behavioral and the ERP index of emotional facial expression (angry, disgust, and happy) were explored.

**Results:** Participants with a higher score of childhood emotional abuse responded faster on the behavioral level and had a smaller P2 amplitude on the neural level when processing disgust faces compared to neutral faces.

**Discussion:** Individuals with a higher level of childhood emotional abuse may quickly identify negative faces with less cognitive resources consumed, suggesting altered processing of emotional facial expressions in young men with a higher level of childhood emotional abuse.

## Introduction

Childhood emotional abuse is broadly defined as the long-time intentional or unintentional inappropriate emotional response and accompanying expressive behavior by caregivers (e.g., verbal abuse, taunting, belittling, and rejection) toward a child (Dottan and Karu, 2006; Norman et al., 2012). The long-term impact of childhood emotional abuse is far less known compared to research on physical abuse and sexual abuse, which is partly because emotional abuse has not been considered as a distinct form of childhood maltreatment until the last few decades (Glaser, 2002; Wright, 2007; Egeland, 2009). It has been shown that emotional abuse is not only a widespread phenomenon but also the core factor underlying different forms of childhood maltreatment (Hart et al., 1997; Iwaniec et al., 2006; Yates, 2007). Childhood emotional abuse has deleterious effects on the behavioral development and mental health of children, as studies found that childhood emotional abuse was associated with a suicidal tendency (de Araujo and Lara, 2016) and eating disorders (Kent et al., 1999; Kennedy et al., 2007; Afifi et al., 2017). By examining the impact of different forms of abuse, studies have found that childhood emotional abuse is more strongly related to depression than physical or sexual abuse (Martins et al., 2014; Nelson et al., 2017). In a longitudinal study, Hamilton et al. (2013) found that adolescence who experienced emotional abuse predicted increased levels of depression and anxiety 9 months later.

Given its emotional characteristics, it could be speculated that individuals who suffered from childhood emotional abuse might show altered emotional function. In the context of chronic childhood emotional abuse, adequately identifying and responding to facial expressions is a crucial and adaptive skill. The explicit recognition and categorization of facial expressions are generally acquired during childhood (Hart et al., 2018). Studies investigating parental rearing indicated that abusive parents exhibit less positive and more negative emotions than non-abusive parents on average (Lyons-Ruth et al., 1987; Kavanagh et al., 1988; Bugental et al., 1990; Curtis and Cicchetti, 2013). In a growth environment full of constant threats to emotional well-being *via* criticism, teasing, or verbal abuse, children might become more sensitive to facial expressions by quickly identifying a threat. Furthermore, Ekman and Friesen (1971) found that the recognition of basic expressions is common across cultures. Therefore, facial expressions are good and comparative research materials. Several studies have shown that childhood abuse has an important impact on facial expression processing (Pollak and Sinha, 2002; McCrory et al., 2011; Garrett et al., 2012; Teicher et al., 2016; Hart et al., 2018; Fang et al., 2019). For example, compared to non-abused controls, abused children (or adolescents/adults who were abused during childhood) exhibited larger biased attention (i.e., faster response) to negative facial expressions (Pine et al., 2005; Romens and Pollak, 2012; Günther et al., 2015; Troller-Renfree et al., 2015; Hart et al., 2018). On the neural level, results from a neuroimaging study showed that adults who reported childhood emotional maltreatment were associated with a significant volume reduction in the left dorsal medial prefrontal cortex (mPFC), the brain region crucial for emotion processing and modulating the limbic system (van Harmelen et al., 2010). In line with this, van Harmelen et al. (2013) found that the childhood emotional maltreatment was associated with the enhanced bilateral amygdala reactivity to both positive and negative emotional facial expressions in general, which they explained that individuals with a history of childhood emotional maltreatment interpret all facial expressions as highly salient. Furthermore, Fonzo et al. (2015) found that the malfunctioned neural circuit of emotion regulation, that is, greater activation of the amygdala and less activation of the right dorsolateral prefrontal cortex (dlPFC) when processing negative facial expressions, mediated the relationship between childhood emotional maltreatment and anxiety symptoms. These previous studies suggested that the relationship between childhood emotional maltreatment and altered emotion processing and/or emotional symptoms might be mediated by a disrupted prefrontal-amygdala circuit.

The event-related potential (ERP) technique, with its high temporal resolution (Hillyard and Kutas, 1983), can allow distinct stages of facial expression processing to be resolved. The N170 component, occurring 130–200 ms subsequent to facial stimuli onset, reflects the structural encoding of facial features and the integration of information on facial identity and expression at an early stage of processing (Eimer, 2011; Norman et al., 2012). The P2 component, with a peak of ~180 ms after stimulus onset, reflects rapid mobilization of attentional resources to salient stimuli (Eimer and Holmes, 2007; Bertsch et al., 2011). The late positive potential (LPP) appears ~400 ms after stimulus onset and is proposed to reflect the sustained allocation of attentional resources to motivationally salient stimuli (Hajcak et al., 2010; Weinberg and Hajcak, 2010). A few studies have explored the impact of different forms of childhood maltreatment on facial expression processing. For example, a larger N170 amplitude to angry faces was found in healthy adults who experienced childhood abuse and neglect than non-maltreated controls (Fang et al., 2019); the P3b amplitude to angry faces was larger in physically abused children than non-abused children (Shackman et al., 2007). On the contrary, James et al. (2018) found that children who received high levels of emotional criticism exhibited smaller LPP to all facial expressions (fearful, happy, and sad) than children who received low levels of emotional criticism from their parents, which they explained that children of critical parents may exhibit avoidance of salient facial stimuli. These mixed results might be due to the different types of maltreatment and different age groups (children vs. adults). Childhood maltreatment has been repeatedly demonstrated linked to changes in brain structure and function from childhood to adulthood (Teicher and Samson, 2016). For example, a longitudinal neuroimaging study found that the childhood maltreatment measured at about 12 years old was associated with retarded growth of the left amygdala in 4 years of the follow-up period (Whittle et al., 2013). Furthermore, most of the previous studies about childhood emotional maltreatment recruited participants with clinical or subclinical psychiatric symptoms, like depression (van Harmelen et al., 2013), or post-traumatic stress disorder (PTSD) (Pine et al., 2005; McCrory et al., 2011; Garrett et al., 2012), which makes it difficult to disentangle the effect of emotional abuse and psychiatric conditions. Therefore, we recruited healthy adults and measured their level of childhood emotional abuse by the Childhood Trauma Questionnaire (CTQ).

Therefore, this study aimed to investigate whether childhood emotional abuse is related to the altered processing of facial expressions^1^ in healthy young men. Based on previous research that individuals with childhood maltreatment would be hypersensitive to emotional facial expressions compared to a non-maltreated group (van Harmelen et al., 2013; Fang et al., 2019), we hypothesized that individuals who had a higher score of childhood emotional abuse would be more sensitive to emotional facial expressions. Specifically, more severity of childhood emotional abuse would be associated with a faster response to negative facial expressions on the behavioral level and greater N170, P2, or LPP amplitudes on the neural level. As an exploratory analysis, we further investigated whether this hypersensitivity is to specific negative facial expressions or all emotional facial expressions.

## Method

### Participants

The sample of this study was restricted to male participants because the current study was part of a large project designed to investigate the psychological and cognitive predictors of the acute stress response, for which saliva cortisol was collected. This study sought to specifically investigate the impact of childhood stress and facial processing. Participants were selected based on the following criterion: (1) no use of any psychiatric, neurological, or endocrine medication; (2) no history of psychiatric, neurological, or endocrine disorder; (3) no history of major chronic physiological disorders; (4) no history of brain injury (such as brain surgery, cerebral hemorrhage, and severe head trauma); (5) no habit of staying up late; (6) no current acute inflammation; and (7) no excessive consumption of alcohol (more than two alcoholic drinks a day) or nicotine (more than five cigarettes a day). Sixty-one male students were recruited from Shenzhen University. One participant was excluded due to the lack of a childhood stress score. In total, 60 participants were included in the final data analysis with a mean age of 19.83 years (SD: 0.93) and mean years of education of 13.47 years (SD: 0.50). All participants provided written informed consent, and they were paid for their participation in the study. This experiment was approved by the Ethics Committee of Human experimentation at the Medical Department of Shenzhen University.

### General Procedure

Participants need filled in the demographic information collection after they arriving at the laboratory. Then, they were put on the elastic cap with electrodes and seated in a dimly lit, sound-attenuated, electrically shielded room. They need completed the gender discrimination task while behavioral and electroencephalography (EEG) data were recorded. Finally, in order to avoid the effect of recall-induced negative emotion on performance, participants completed the Negative Affect Scale (NAS) and the Emotional Abuse Subscale from CTQ as described in the Questionnaires section after completing the experiment.

### Stimuli

The stimuli consisted of photographs of four actors (two women), which were taken from the Chinese Facial Expressions of Emotion (Wang and Markham, 1999). Each actor displayed a happy, neutral, angry, and disgust expression. A total of 16 photographs were used in the experiment. All photographs matched for luminance and contrast. Participants were seated with their eyes ~70 cm from the computer screen. All facial stimuli presented were shown on a black background and subtended a viewing angle of 7.7 × 6.3°.

### Gender Discrimination Task

**Figure 1:**
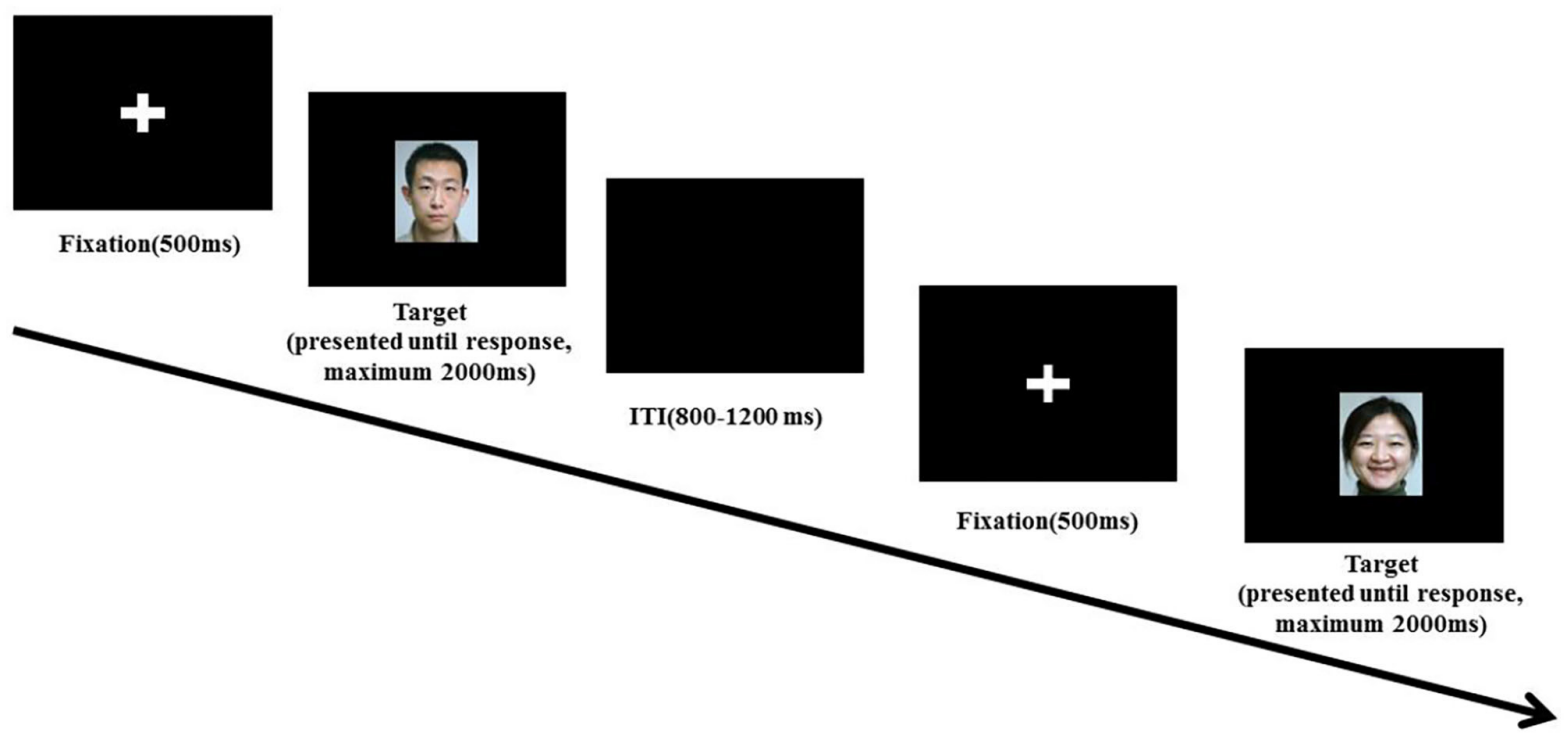
Schematic illustration of the experimental procedure.

To direct the attention of participants to the stimuli but not to consciously identify the facial expressions, the gender discrimination task was adapted from a previous study (Sprengelmeyer and Jentzsch, 2006). After an initial practice block of five trials, one experimental block of 160 trials was completed. Each facial expression was presented 10 times in a pseudorandom order; thus, the consecutive presentation of the same facial expressions was avoided. As shown in Figure 1, each trial started with a 2 × 2 cm white fixation cross at the center of the screen for 500 ms. After the fixation cross, a facial expression was presented until the participants responded in the time window of 2,000 ms. Participants were asked to respond to the gender of the facial stimuli as fast and accurately as possible. The inter-trial interval (ITI) was 800–1,200 ms. To balance the possible difference between the response of right and left hands, half of the participants were instructed to press the left key when male faces were presented and the right key when female faces were presented, the other half received the reverse instructions. Participants pressed the left and right keys by index fingers of their left and right hands, respectively.

### Questionnaires

Childhood emotional abuse was assessed by Emotional Abuse Subscale from CTQ on a 5-point Likert scale (1, never true; 5, very often true) (Bernstein and Fink, 1998). The CTQ has been used as a quantitative measure of the severity of childhood adversity in different populations with or without psychopathology (Viola et al., 2016). The Chinese version of CTQ has been demonstrated good internal consistency and validity in healthy samples (Zhao et al., 2005). The sample items of emotional abuse are: “When I was growing up, people in my family said hurtful or insulting things to me,” and “People in my family called me things like stupid, lazy, or ugly.”

Negative mood state was assessed by NAS on a 5-point scale from 1 (not at all) to 5 (very much) (Watson et al., 1988; Huang et al., 2003). NAS was used as a covariate in the data analysis to correct for possible recall bias, which is common among individuals who suffered from traumatic events (Amir et al., 1996; Tapia et al., 2012). Previous studies found that negative mood was associated with negative recall bias (Bradley and Mogg, 1994; Raphael and Cloitre, 1994; Holland and Kensinger, 2010). In turn, this recall bias might result in an overestimation of childhood stress.

### EEG Recording and Preprocessing

According to the international 10–20 system, the EEG was recorded by Ag/AgCl electrodes mounted on 64 scalp sites in the elastic cap (Neuroscan Inc., Charlotte, North Carolina, USA). The online reference was the left mastoid and the data were referenced offline to the average of both mastoids. Vertical eye movement was recorded by one pair of electrodes placed above and below the left eye and horizontal eye movement was recorded by another pair placed 10 mm from the outer canthi of each eye. The impedance from all electrodes was below 5 KΩ. Signals were amplified with a bandpass filter at 0.05–100 Hz and digitized at 1000 Hz. The EEG data were preprocessed by Scan 4.3 software (Neuroscan, USA). The ocular artifacts were removed from the EEG signal by a regression procedure implemented in the Neuroscan software (Semlitsch et al., 1986). Trials with artifacts were automatically rejected with a criterion of ± 100 μV. The data were filtered with a 30 Hz lowpass filter.

The EEG data were epoched into periods of 1,000 ms (including 200 ms before stimulus onset as baseline) and time-locked to the onset of the stimulus. For the present experiment, N170, P2, and LPP components were measured and analyzed. For the N170, the mean amplitude was analyzed between 150 and 180 ms after stimulus onset at bilateral parietal-occipital sites (P7 and P8). The mean amplitude of P2 was obtained at the frontal-central sites (F3, Fz, and F4) with a time window of 145–175 ms. For the LPP, the mean LPP amplitude was obtained at parietal electrodes (CP3, CPz, and CP4) with a time window between 350 and 600 ms. These sites and time windows were chosen according to the data where they have the maximum amplitude and are also in line with previous research (Cuthbert et al., 2000; Huang and Luo, 2006; Luck and Kappenman, 2013; Zhang et al., 2016). To reduce statistical error, the ERP components (N170, P2, and LPP) were averaged across their respective electrodes.

### Data Analysis

For the behavioral performance, repeated measures ANOVAs were used to investigate the effect of facial expressions (angry, happy, disgust, and neutral) on accuracy and reaction time (RT) in the gender discrimination task. Trials with RT below 100 ms and above 2,000 ms were excluded from behavioral and ERP analyses.

To detect the effect of the facial expressions on the ERP components (N170, P2, and LPP), repeated measures ANOVAs were first conducted on facial expressions (angry, disgust, happy, and neutral). To reduce the interference of the physical characteristics of visual stimuli (Kujawa et al., 2016), the behavioral and ERP data were further calculated as the difference value (Δ). More specifically, the emotional effect for behavioral performance (RT and accuracy) was measured as the RT/accuracy to emotional facial expressions (angry, disgust, and happy) minus the RT/accuracy to neutral facial expressions. Similarly, the emotional effect for ERP components was also calculated by the difference value of ERP components between the emotional facial expressions and neutral facial expressions. The Greenhouse-Geisser correction for degrees of freedom was applied when the sphericity assumption was violated.

To explore the possible relationship between childhood emotional abuse and dynamic stages of emotion processing, associations between the score of emotional abuse and ΔRT/accuracy and ΔERP amplitude by emotional facial expressions (angry, disgust, and happy) were examined by using the Pearson correlation analyses. To control for the multiple comparisons problem, the corrected *p-*value was set at 0.017.

To further characterize the influence of emotional abuse on the dynamic stage of emotional processing in adulthood, we conducted multiple regression analyses on the significant correlations separately. In the regression model, the ΔRT/accuracy and ΔERP amplitude were treated as dependent variables and emotional abuse score as the independent variable. Since age, general intelligence, and mood state may have a potential influence on facial expression processing (Amir et al., 1996; Tapia et al., 2012; Lewis et al., 2016; Vesker et al., 2018; Connolly et al., 2019; Durbin et al., 2019), the demographic (age and education years) and current mood state (NAS) were treated as covariate variables. Considering that the Bootstrap method provides better control over type I error and a better representation of the probability distribution and can be used to analyze non-normal distribution data (Wilcox, 2012), we performed multiple regression with bootstrapping (*n* = 1,000 times of resampling with replacement) to improve the robustness of the inference. Results from the regression analysis are described as regression coefficients with a 95% CI and SEs from the bootstrap analysis.

Data were analyzed with the Statistical Package for the Social Sciences (SPSS 25.0 IBM Corp., Armonk, NY, USA).

## Results

### Subjective Measurements

The mean score of emotional abuse was 7.25 (SD: 2.31 range: 5.00–17.00). The mean score of NAS was 14.20 (SD: 5.01, range: 10.00–33.00).

### Behavioral Performance

The accuracy (%) analyzed to angry, disgust, happy, and neutral faces were 97.29 (±2.97), 97.52 (±2.96), 97.50 (±2.98), and 97.83 (±2.92), respectively. The RT (ms) analyzed to angry, disgust, happy, and neutral faces were 523.70 (±101.06), 517.68 (±92.70), 523.14 (±103.17), 520.83 (±97.10), respectively. Participants performed quite well in this experiment, and the overall accuracy rate was 97.52% (SD: 2.95%). The repeated measures ANOVAs revealed the main effect of facial expressions on accuracy (*F*_(3,177)_ = 0.66, *p* = 0.58) and RTs (*F*_(3,177)_ = 1.35, *p* = 0.19) was not significant. Observing a potential ceiling effect for accuracy, we mainly focused on the RT. The ΔRT for angry faces was 2.88 (SD: 21.72); for disgust faces was −3.14 (SD: 25.51); and for happy faces was 2.31 (SD: 24.77).

### Event-Related Potentials

**Figure 2:**
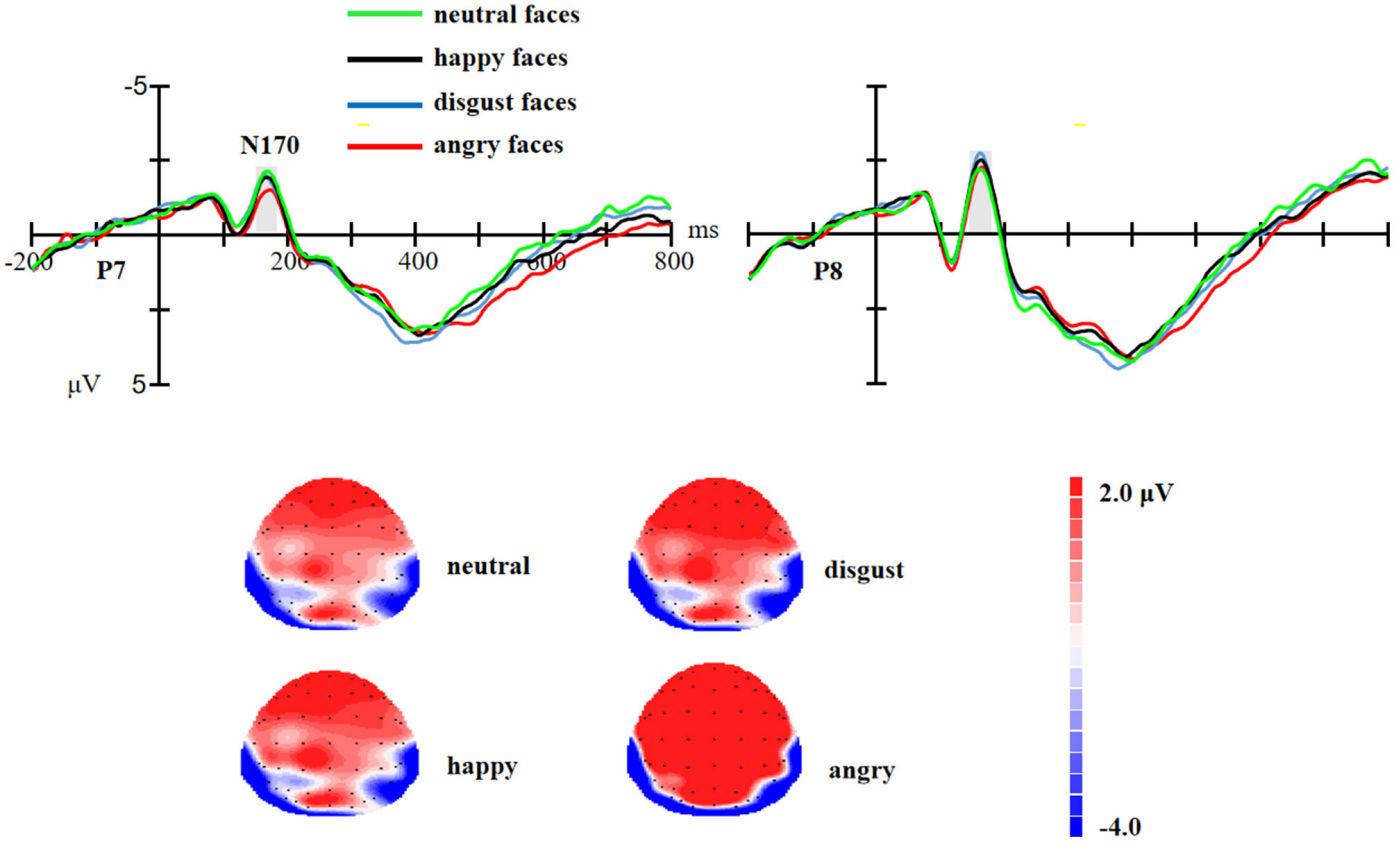
Upper: The event-related potential (ERP) waveforms for angry, disgust, happy, and neutral facial expressions at P7 and P8 electrode sites. Bottom: The scalp distribution of the mean N170 amplitude (150–180 ms) for angry, disgust, happy, and neutral facial expressions.

For the N170 component, repeated ANOVA showed that there was no significant effect of the facial expressions on the N170 amplitude [*F* (3,174) = 1.31, *p* = 0.26, η^2^ = 0.02, as shown in Figure 2].

**Figure 3:**
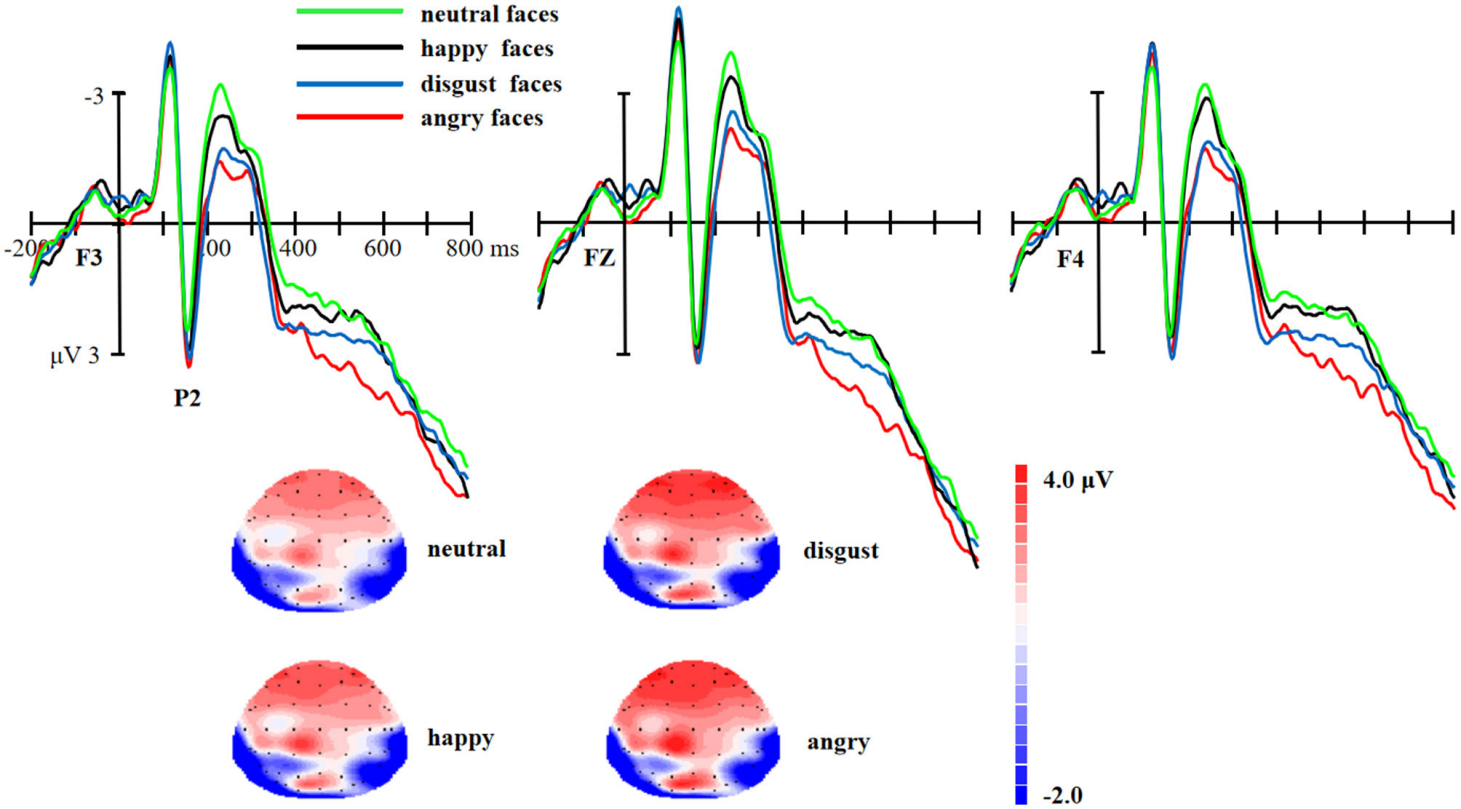
Upper: The ERP waveforms for angry, disgust, happy, and neutral facial expressions at F3, FZ, and F4 electrode sites. Bottom: The scalp distribution of the P2 (145–175 ms) for angry, disgust, happy, and neutral facial expressions.

As for the P2, there was a marginally significant effect of facial expressions [*F* (3,174) = 2.25, *p* = 0.08, η^2^ = 0.04]. *Post-hoc* tests revealed that the P2 amplitudes were larger for angry faces (*p* = 0.04) and disgust faces (*p* = 0.03, as shown in Figure 3) than neutral faces. No significant difference was found between angry and disgust faces (*p* > 0.05) or between happy and neutral faces (*p* > 0.05).

**Figure 4:**
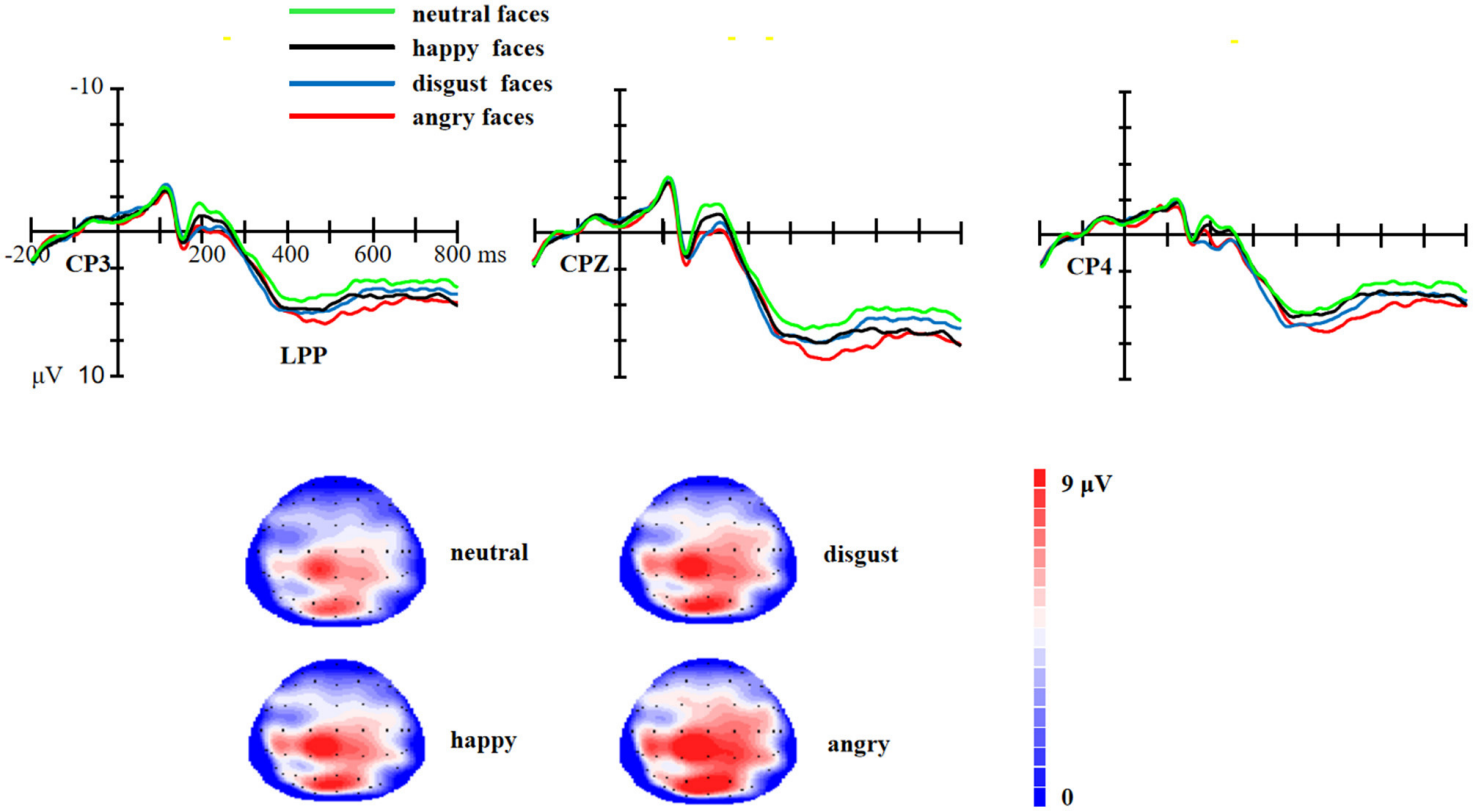
Upper: The ERP waveforms for angry, disgust, happy, and neutral facial expressions at CP3, CPZ, and CP4 electrode sites. Bottom: The scalp distribution of the LPP (350–600 ms) for angry, disgust, happy, and neutral facial expressions.

As for the LPP, there was a significant effect of facial expressions [*F* (3,177) = 12.23, *p* < 0.01, η^2^ = 0.17]. *Post-hoc* tests revealed that the LPP amplitudes were larger for happy faces (*p* < 0.01), angry faces (*p* < 0.01), and disgust faces (*p* < 0.01, as shown in Figure 4) than for neutral faces. The angry faces elicited a larger LPP than disgust faces (*p* = 0.04) and happy faces (*p* < 0.01). There was no significant difference between disgust faces and happy faces (*p* > 0.05).

In summary, the main effect of emotional facial expressions was only found on the P2 and LPP components. Therefore, for the following correlation analyses, we focused on these two components, and the ΔP2 and ΔLPP amplitudes were calculated by the amplitude of emotional facial expressions (angry, disgust, and happy) minus the amplitude of neutral facial expressions.

### The Relationship Between Childhood Stress and Dynamic Stages of Facial Expression Processing

There was a negative relationship between the emotional abuse score and the ΔRT for disgust faces (*r* = −0.32, *p* = 0.013). The relationships between the emotional abuse score and the ΔRT for angry (*r* = −0.19, *p* = 0.14) and happy (*r* = −0.04, *p* = 0.76) facial expressions were not significant.

The emotional abuse score was negatively correlated with the ΔP2 amplitude for disgust facial expressions (*r* = −0.31, *p* = 0.017). However, the correlation coefficient between the emotional abuse score and the ΔP2 amplitude for angry (*r* = −0.10, *p* = 0.48) and happy (*r* = −0.01, *p* = 0.92) facial expressions did not reach significance.

As for the ΔLPP amplitude, the relationships between the emotional abuse score and the ΔLPP amplitude for disgust, angry, or happy facial expressions did not reach significance (|r|s = 0.02–0.10, *p* > 0.05).

Since the ΔRT and ΔP2 both passed the multiple comparisons in the correlation analysis, and to explore the predictive value of childhood adversity on dynamic stages of emotional facial expressions in adulthood, more strict multiple regression with bootstrapping was applied for the significant correlations.

**Figure 5:**
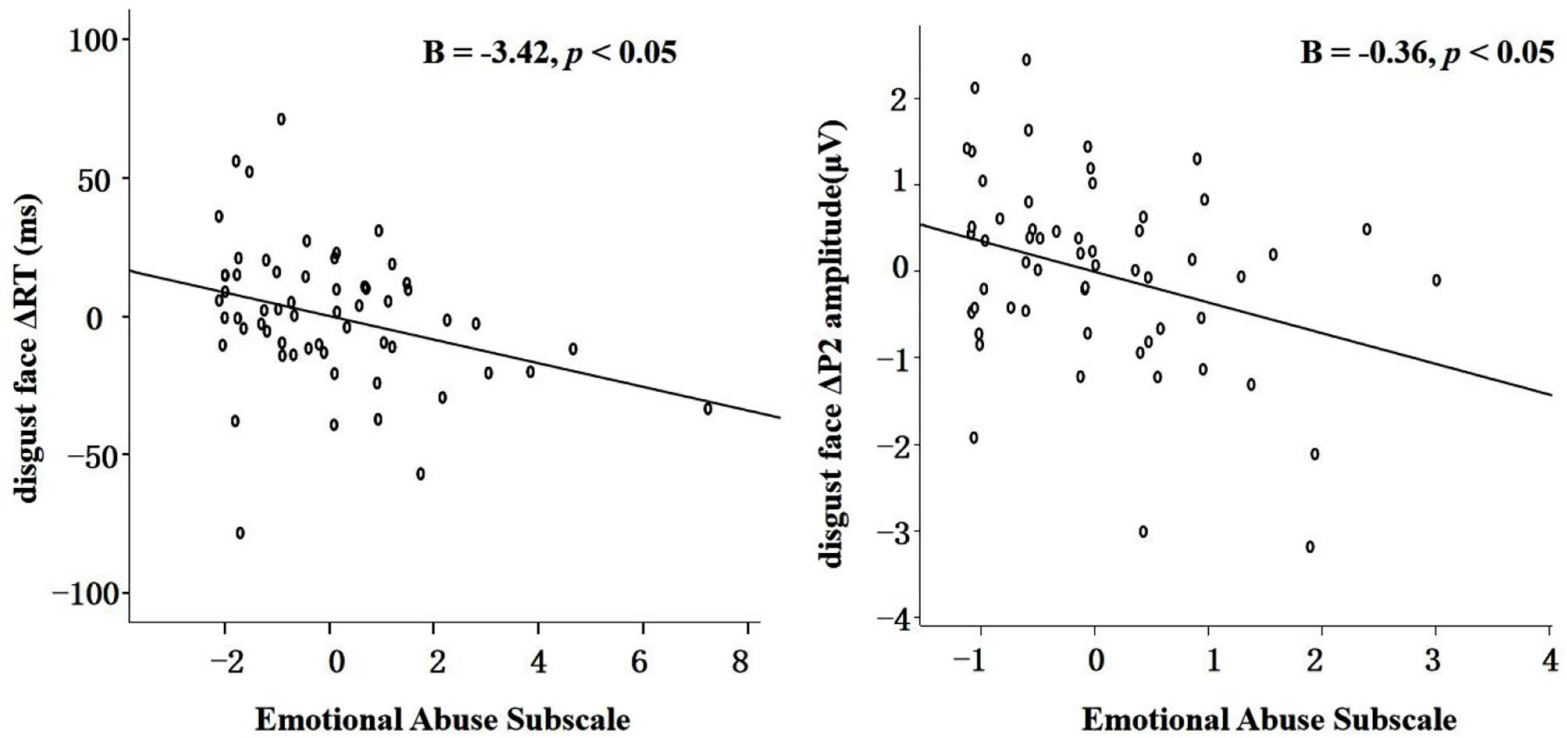
Left: The partial correlation scatterplot of the relationship between emotional abuse score and the ΔRT for disgust faces (RT for disgust faces minus RT for neutral faces). Right: The partial correlation scatterplot of the relationship between emotional abuse score and the ΔP2 amplitude for disgust faces (the P2 amplitude for disgust faces minus the P2 amplitude for neutral faces).

For the model of ΔRT for disgust faces [*R*^2^ = 0.10, *F* (4,58) = 1.55, *p* > 0.10, as shown in Table 1], only emotional abuse score significantly negatively predicted the RT (*B* = −3.42, 95% CI: −6.16 to −0.89, SD: 1.27, *p* = 0.01, as shown in Figure 5 Left).

**Table 1:** Bootstrapping regression of emotional abuse on the ΔRT for disgust faces.

	**Unstandardized regression coefficient B**	**95% CI**	**SE (bootstrap)**	***p***
Age	0.44	−8.42 to 7.51	3.99	0.91
Education years	−2.29	−15.19 to 13.35	7.18	0.74
NAS	0.06	−0.68 to 1.19	0.49	0.89
Emotional abuse	−3.42	−6.16 to −0.89	1.27	0.01

*NAS, Negative Affect Scale represents the score of current negative affect*.

For the model of ΔP2 amplitude for disgust faces [*R*^2^ = 0.13, *F* (4,57) = 1.93, *p* > 0.10, as shown in Table 2], only emotional abuse score significantly negatively predicted the ΔP2 amplitude (*B* = −0.36, 95% CI: −0.75 to −0.06, SD: 0.18, *p* = 0.04, as shown in Figure 5 Right).

**Table 2:** Bootstrapping regression of emotional abuse on the ΔP2 amplitude for disgust faces.

	**Unstandardized regression coefficient B**	**95% CI**	**SE (bootstrap)**	***p***
Age	0.50	−0.19 to 1.13	0.32	0.12
Education years	−0.57	−0.95 to 0.87	0.64	0.38
NAS	−0.01	−0.09 to 0.08	0.04	0.84
Emotional abuse	−0.36	−0.75 to −0.06	0.18	0.04

*NAS, Negative Affect Scale represents the score of current negative affect*.

### Explorative Analysis

To explore whether emotional abuse is associated with the altered processing of specific facial expressions, we further compared the correlation coefficients among different facial expressions. The test computational method for the comparison of the correlation coefficients is a *z*-test on the Fisher *z*-transformed correlation coefficients (Hinkle and Wiersma, 1988). The result showed that the correlation between the emotional abuse score and the ΔP2 amplitudes for disgust facial expressions was marginally larger than the correlation between the emotional abuse score and the ΔP2 amplitudes for happy facial expressions (*z* = −1.63, *p* = 0.05). However, there was no significant difference in correlation coefficients between the emotional abuse score and the ΔP2 amplitudes for disgust facial expression and angry facial expression (*z* = −1.17, *p* = 0.12). These results showed that childhood emotional abuse is not only related to altered processing for disgust facial expressions but also for angry facial expressions, which suggested that childhood emotional abuse might be associated with disrupted processing of negative facial expressions but intact positive facial expression processing.

## Discussion

The present study investigated the relationship between the severity of childhood emotional abuse and the processing of facial expressions in healthy young males. There are two major findings in this study: (1) on the behavioral level, higher severity of childhood emotional abuse was associated with faster response to disgust faces compared to neutral faces; (2) on the neural level, higher severity of emotional abuse was associated with the smaller P2 amplitude for disgust faces compared to neutral faces. These findings were demonstrated in healthy males without any current or history of psychiatric disorders, and the potential confounding factors of age and education and the current negative mood were controlled.

On the behavioral level, we found that higher severity of childhood emotional abuse was associated with a faster reaction time to disgust faces. This result echoed previous findings that participants who were maltreated during childhood responded faster to negative facial expressions (e.g., fearful and sad) compared to non-maltreated adolescents (Günther et al., 2015; Hart et al., 2018). On the neural level, emotional abuse was correlated with the smaller P2 amplitude for disgust faces. The P2 component reflects the rapid detection of salient information or the rapid mobilization of attentional resources (Eimer and Holmes, 2007; Bertsch et al., 2011). In the so-called threat detection-response circuit, the amygdala is proposed to be modulated by both a cortical, conscious route (prefrontal appraisal processes) and an entirely subcortical, non-conscious route in which the salient stimuli directly project to the amygdala without prefrontal assessment (LeDoux, 1996; LeDoux and Penguin, 2003). A few researchers found that compared to non-maltreated children, maltreated children had an increased amygdala activation to subliminally presented negative emotional faces in which the more-rapidly engaged, non-conscious component from “threat detection-response circuit” is likely to predominate (Garrett et al., 2012; Dannlowski et al., 2013). Furthermore, an increased amygdala activation to subliminally presented negative emotional faces was also found in childhood-maltreated adults without psychopathology (Bogdan et al., 2012; Dannlowski et al., 2013). Therefore, individuals with higher severity of childhood emotional abuse are likely to detect and respond faster to negative emotional faces by the fast, automatic, non-conscious amygdala processing.

In an emotionally abusive environment, fast detection of threatening facial expressions may help a child to avoid confrontation with the offender. This adaptive response may lead to experience-specific information processing bias, that is, persistent hypervigilance for negative facial expressions in the long run (Gibb et al., 2009). With a continuous image transformation procedure, Pollak and Sinha (2002) found that physically abused children can accurately identify angry facial expressions by less sensory input than controls, indicating that the ability to recognize threatening facial expressions is facilitated by the perceptual hypersensitivity in abused children. The P2 component, as one part of a cognitive-matching system, is related to comparing sensory inputs with stored memories (Luck and Hillyard, 1994). Therefore, the smaller P2 amplitude in emotionally maltreated participants might be due to the quick match between the perceived negative faces and their stored memory templates of negative facial expressions shaped by early life experience. Therefore, the relationship between the emotional abuse and the attenuated P2 amplitudes and faster response to disgust facial expressions might be mediated by biased attention to negative emotions, i.e., participants with a higher score of childhood emotional abuse may automatically identify negative faces with a less cognitive resource. It is worthwhile to mention that the emotional abuse score in this study is mostly low to moderate, and all the participants are relatively healthy. The result might be explained from the perspective of resilience or adaptation to childhood adversity rather than vulnerability.

Exploratively, we found that the correlation between childhood emotional abuse and neural processing for disgust facial expressions was not different from the correlation for angry facial expressions. This suggested that childhood emotional abuse might be related to heightened sensitivity with a low level of specificity to negative facial expressions. This result was also echoed with previous findings that individuals with maltreatment histories have an abnormal reaction to emotional faces, particularly those seen as threatening (for a review, refer Teicher et al., 2016). According to previous literature, disgust facial expressions are related to expulsion, which is a threat to self-identity (Phillips et al., 1998; Nicol et al., 2014); whereas angry facial expressions are often seen as aggressive, which is a threat to physical integrity (Pollak and Tolley-Schell, 2003). Adults who grew up in abusive situations can selectively and preferentially process signals of threat, in turn, facilitate threat detection. However, this result cannot equal the finding of a positive effect of processing angry faces and childhood emotional abuse. To prove this hypothesis, we may need a larger sample size and richer sample population (e.g., participants with a severe history of child emotional abuse) in future research.

There are several limitations to this study. First, only male undergraduates were recruited. On the one hand, this specific sample mitigates potential confounding factors such as hormonal contraceptive use or menstrual cycle. On the other hand, considering that childhood abuse might influence mental health in a gender-specific manner (Abrams, 2019), the results might not be able to generalize to the female population. Second, we did not preselect the participants with severe emotional abuse experience, and all the participants are healthy. Therefore, it is not certain whether the result of hypersensitive facial processing reflects a risk or rather a resilient factor to future stress. Third, the sample size in the current study was relatively small, which might limit the power of our results. To make the results more reliable, we used the bootstrapping procedure with a stricter significant value (0.017) in the statistical analysis, which we expected to compensate for the small sample size to some extent. Fourth, we only used the self-reported questionnaire to measure childhood emotional abuse. Though we controlled the current negative mood to avoid the recall bias on traumatic events, it would be better to confirm the severity of emotional abuse by both self-report measures and interviews. Finally, more emotional facial expressions (such as fearful and sad) and more sophisticated paradigms should be considered in future research.

## Conclusion

In summary, we found that a higher score of childhood emotional abuse was associated with both faster response on the behavioral level and the attenuated P2 amplitude on the neural level to negative faces, suggesting that individuals with a higher level of childhood emotional abuse may automatically identify negative faces with a less cognitive resource. Such alterations might, on the one hand, increase survival probability by efficient threat processing. On the other hand, this emotional hypersensitivity might become a risk factor for developing psychiatric disorders after exposure to another major stressor in the future like depression and anxiety (Ferguson and Dacey, 1997; Bifulco et al., 2002).

## Data Availability Statement

The original contributions presented in the study are included in the article/supplementary material, further inquiries can be directed to the corresponding author.

## Ethics Statement

The studies involving human participants were reviewed and approved by Ethics Committee of Human experimentation at the Medical Department of Shenzhen University. The individual(s) provided their written informed consent for the publication of any identifiable images or data presented in this article.

## Author Contributions

YL: statistical analysis, manuscript preparation, manuscript defnition of intellectual content, and data acquisition. HD: study concepts, manuscript defnition of intellectual content, manuscript editing, manuscript revision/review, and manuscript final version approval. JW: guarantor of integrity of entire study, study design, and study concepts. HP: study concepts, study design, data acquisition, and data analysis/interpretation. All authors contributed to the article and approved the submitted version.

## Funding

This work was supported by the National Natural Science Foundation of China (31771246, 31920103009, 31871115); Shenzhen-Hong Kong Institute of Brain Science-Shenzhen Fundamental Research Institutions (2021SHIBS0003).

## Conflict of Interest

The authors declare that the research was conducted in the absence of any commercial or financial relationships that could be construed as a potential conflict of interest.

## Publisher's Note

All claims expressed in this article are solely those of the authors and do not necessarily represent those of their affiliated organizations, or those of the publisher, the editors and the reviewers. Any product that may be evaluated in this article, or claim that may be made by its manufacturer, is not guaranteed or endorsed by the publisher.

## Footnotes

^1^The facial expressions that we chose here are positive (happy), negative (angry and disgust), and neutral. As for the negative emotions, angry and disgust belong to different dimension, as angry is associated with aggression, whereas disgust is associated with rejection (Schlosberg, 1954; Kuppens et al., 2003; Olatunji and Sawchuk, 2005). According to the previous research, emotionally abusive care-givers might be more likely to show dislike (such as calling “ugly”) or aggression (such as criticism) (Bernstein and Fink, 1998; Finzi-Dottan and Karu, 2006; Francis and Wolfe, 2008). Therefore, the aim of the study was to explore how the level of emotional abuse relates to processing facial expressions, especially those seen as threatening.
